# Symptoms 6 months following SARS-CoV-2 infection in Nepali women

**DOI:** 10.1371/journal.pone.0299141

**Published:** 2024-03-11

**Authors:** Deepak S. Shrestha, Sajani Manandhar, Bimal Sharma Chalise, Sagar Kumar Rajbhandari, Anup Bastola, Parmananda Bhandari, Santa Kumar Das, Pankaj Pant, Sangita Sharma, Hari Prasad Kattel, Roshan Kumar Jha, Mahendra Raj Shrestha, Anil Shrestha, Richard R. Love

**Affiliations:** 1 Department of Internal Medicine, People’s Dental College and Hospital, Kathmandu, Nepal; 2 New ERA, Kathmandu, Nepal; 3 Sukraraj Tropical and Infectious Disease Hospital, Kathmandu, Nepal; 4 Tribhuvan University Teaching Hospital, Kathmandu, Nepal; 5 Nepal Armed Police Forces Hospital, Kathmandu, Nepal; 6 Independent researcher, Madison, United States; Federal Medical Centre Abeokuta, NIGERIA

## Abstract

In Nepal, over 1 million individuals have tested positive for SARS-CoV-2. We sought to describe the frequency of nonrecovery from this infection at 6 months and associated symptoms. We conducted a retrospective cohort study of 6142 women who had positive and negative PCR tests for this infection 6 months previously at 3 institutions in Kathmandu. In telephone interviews women provided information on 22 symptoms and their intensities, health status and history, and functional status. Of 3732 women who had tested PCR positive, 630 (16.9%) reported that they were unrecovered. These 630 unrecovered women were distinguished statistically from the 3102 recovered women by more frequent histories of allergies, rheumatoid disease, BCG immunization, Covid vaccination, strep throat and recent URIs, and both weight gain and weight losses of more than 5 kg in the 6 months following testing, and stressful events in the preceding year. Fatigue, pain, difficulty remembering, shortness of breath, heat and cold intolerance and unrefreshing sleep were reported in 41.9% to 10.5% of these 630 unrecovered women. Six months after confirmed SARS-CoV-2 infection 16.9% of Nepali women have long-COVID manifested as an immune, metabolic, and hormonal systems disruptive and dysfunction syndrome

## Introduction

In Nepal, over 1 million individuals have tested positive for SARS-CoV-2, and this figure may underestimate actual numbers of cases because of limited testing. Data in patients beyond 3 months from diagnosis of this infection about symptoms, their severities, and timelines of these, are limited, particularly in low- and middle-income countries. In high-income countries, investigative journal reports have suggested that 10 to 30 percent of infected individuals, more commonly middle-aged women, have persistent functional capacity-limiting symptoms, 6 months and beyond the time of initial diagnosis [1–4].

Persistent symptoms following Covid infection, mimic post-infectious disease syndromes reported for multiple viral illnesses, Lyme disease, infectious mononucleosis, viral hepatitis, Q fever and SARS-1, as well as those of chronic fatigue syndrome (CFS)- Myalgic Encephalomyelitis (ME), a poorly understood, complex and chronic clinical syndrome affecting women four times more often than men [5–14]. CFS/ME is characterized by at least 6 months of mental and physical fatigue, muscle weakness exacerbated by physical and social/mental exertion, malaise, pain, non-restorative sleep, and cognitive impairment [14]. CFS/ME is a clinical diagnosis, with neuroinflammatory, metabolic, and hormonal physiological features [15].

Because of the significant health, social and economic consequences of persistent symptoms following SARS-CoV-2 infection, we designed a study to describe the frequencies and intensities of the commonest reported symptoms, health history correlates and functional status in a convenience sample of women tested for this infection because of suggestive symptoms 6 months previously, in order to evaluate similarities of suggested non-recovery from this infection to Chronic Fatigue Syndrome/Myalgic Encephalomyelitis [14].

## Methods

We conducted a retrospective cohort study of women who, between August 3, 2020, and September 29, 2021, had self-referred themselves and then underwent PCR tests for SARS-CoV-2 done at three referral institutions in Kathmandu (Sukraraj Tropical and Infectious Disease Hospital, Tribhuvan University Teaching Hospital and Nepal Armed Police Forces Hospital) because they were symptomatic with fever, shortness of breath, cough, or anosmia. Between March 21, 2021, and March 24, 2022, trained Nepali interviewers who were unaffiliated with the testing institutions, called 6481 consecutive test-positive case women approximately 6 months (minimum 4 months) following their PCR test. If a family member answered the call and reported that the individual had died, this was recorded. Three attempts were made to contact these women. Participants were briefed about the study, as explained in the ethics committee-approved protocol, informed verbal consent sought with no incentive offered for participating in the study, before enrolling them in the study.

**Fig 1:**
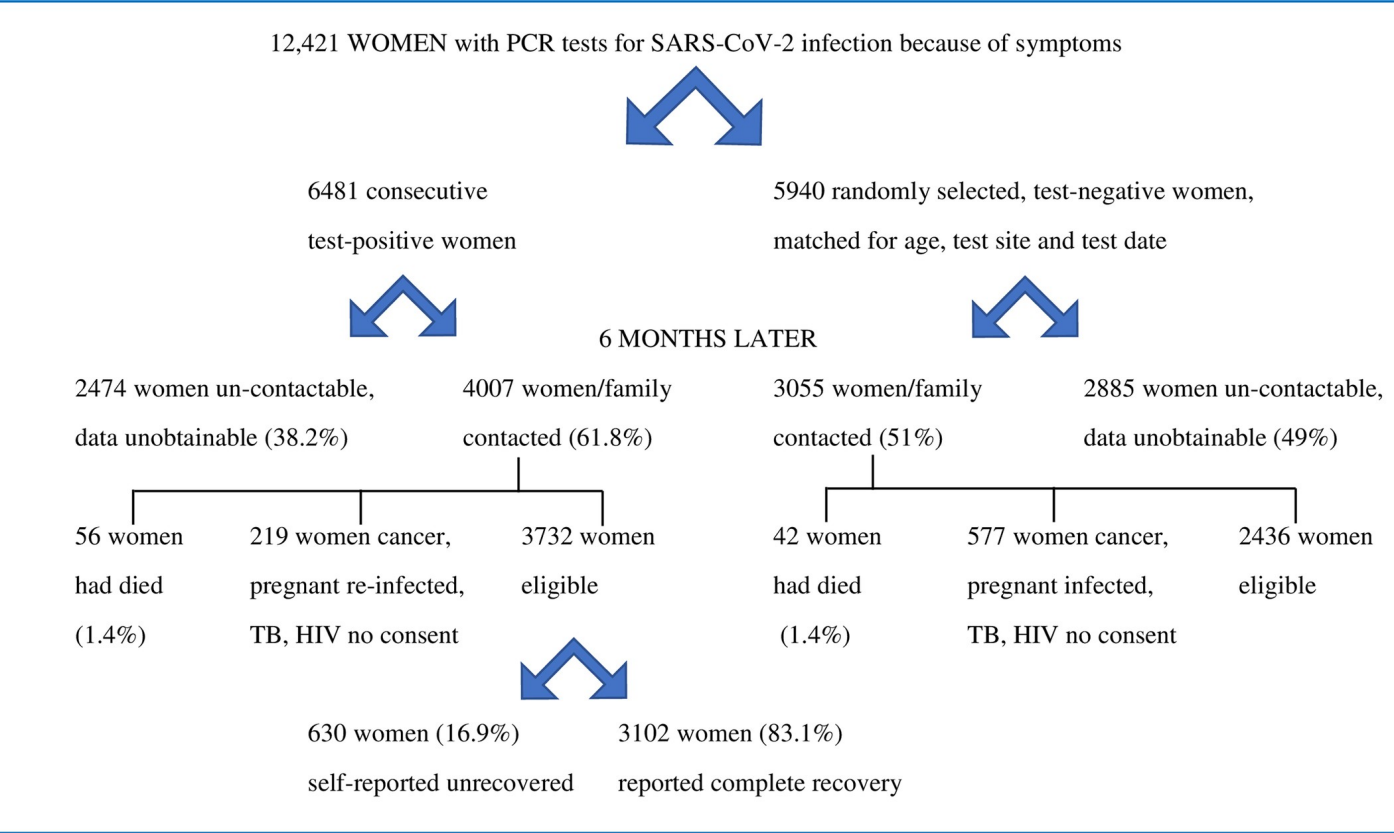
Consort diagram of study populations.

During the same study period, attempts were made to call 5940 randomly selected age (in same 5-year age group) and test date-matched women who had tested negative for SARS-CoV-2 at the same institutions 6 months previously (Fig 1).

All successfully contacted and consented case (test-positive) and control (test-negative) women were first screened for histories of reinfections (test positive cases) or infections (test negative controls) with SARS-CoV-2, cancer, tuberculosis, pregnancy, mental illness, and HIV. Individuals who did not report these medical conditions were study eligible. Interviewers then asked participants about their current symptoms, health status history and functional status. The development and validation of the symptom questionnaire has been reported in another publication [16]. The functional status questions were selected from among 90 items of the Common data elements for evaluation of Chronic Fatigue Syndrome/Myalgic Encephalomyelitis by the United States National Institutes of Health [17]. A question about Covid-19 vaccination asked whether the patient had ever had such immunization, details of which were not ascertained; The Chinese Sinopharm BIBP, AstraZeneca, and Johnson and Johnson immunizations were available to these women. Any positive response would very likely have indicated receipt of a vaccine after the testing date report which anchored entry into the study.

For the patients who had tested positive, we then asked about their recovery. “Do you feel that you have not completely recovered from your Covid-19 infection?” followed by other questions seeking confirmatory or clarifying responses about “feeling unwell” attributed to Covid-19 infection and feeling unreturned to usual level of health following Covid-19 infection. We defined a subgroup of test-positive cases as “unrecovered from SARS-CoV-2 infection” based on affirmative response to this questioning.

We used Pearson’s chi square or Fisher’s exact test for categorical data, and mood’s median test for continuous data in comparisons of characteristics between groups. All p values were two sided; p values of <0.05 were considered statistically significant. Statistical analyses were done with SPSS version 25.

The study was approved by the Ethics Committee of the Nepal Health Research Council I.D. # l8l / 202l, on 12 March 2021, and amended on 24^th^ August 2021, and subsequently the Institutional Review Board at Marquette University in the United States. The senior author (RRL) provided the funding for the research and was involved in all aspects of this activity as indicated in the section detailing authors’ contributions.

## Results

The test positive (42.4%) and test negative (59%) potential study subjects who were not study eligible occurred because for the majority of these no contact could be made after 3 phone calls, attributed to: no answer, wrong number, left the country, number change and new number unknown, or among significant numbers of potential subjects, the consent and data-obtaining processes were unimplementable because of the associated time burdens. The greater percentage of test negative individuals who were not study eligible occurred because 7.5% of identified subjects had subsequently developed SARS-CoV-2 infection, and a larger percentage of these individuals refused to acknowledge the subject of the call. The age, test site, test date characteristics of the study eligible and ineligible subjects were similar.

Among the 3732 test-positive study eligible women (Median age 35 years, age range: 18–96 years; interview timepoint from diagnosis range 4–8 months, median 6.0 months, mean 5.7 months); 630 (16.9%) (Median age 37 years, age range: 18–77 years, interview timepoint from diagnosis range 4–8 months, median 6.0 months, mean 5.7 months) reported that they had not completely recovered from their SARS-CoV-2 infection.

The 2436 test negative study eligible women had similar demographic characteristics: (Median age 35 years, age range: 8–82 years, interview timepoint from diagnosis range 3.3–9.6 months, median 6.0 months, mean 6.4 months). The 630 unrecovered women differed statistically from the recovered and the test negative women with greater median age (37 versus 35 and 35 years) (p = 0.002, and p = 0.03).

Table 1 shows the characteristics of the three study populations. Unrecovered women are statistically different from the recovered women with more frequent histories of allergies, asthma, BCG immunization, rheumatoid disease, strep throat, and URIs in the preceding year. Unrecovered women were statistically different also from recovered women in reporting both weight gain and weight losses of more than 5 kg in the 6 months after SARS-CoV2 testing, higher usual (before illness) level physical activity which may suppress hormonal fluctuation, and more frequent stressful events in the preceding year. Age, diabetes, tobacco abuse, hypertension history, and BMI statistically significant differences were not observed between these two groups.

**Table 1:** Demographic and Medical History Characteristics among 3 groups of women: 1) Test positive women self-assessed as unrecovered, 2) Test positive women self-assessed as recovered, and 3) Test negative women who had remained Covid-19 symptoms free.

Demographic and Medical History Characteristics	Group 1 Test + Unrecovered	Group 2 Test + Recovered	Group 3 Test–and Disease-Free
Total	630	3102	2463
Time from PCR test (mean months)	5.7	5.7	6.4
Mean age (years)	37.4	37	37.1
Median age ^b^^,^ ^c^ (years)	37	35	35
Patients aged 50 and > (%)	15.7	16.9	15.6
Age range (years)	18–77	18–96	18–92
Calculated BMI (kg/m^2^)	25.3	25	24.9
BMI > = 30 (%)	13.1	13.1	11.8
BMI> = 25 (%)	50.4	48.2	46.2
Pregnancy in year before Covid test (% yes)^f^	2.3	3.3	5.9
Pregnancies (mean #)	1.4	1.4	1.5
Menstrual period in preceding 3 months before interview (% yes) ^d^	77.3	73.9	71.4
Weight increases by 5 or more kg since testing (% yes) ^e^^,^ ^f^	16.8	8.9	10.6
Weight decreases by 5 or more kg since testing (% yes) ^e^^,^ ^f^	10.6	4.5	6.2
Hypertension history (%) ^c^	12.7	10.2	9.9
Diabetes history (%)	4.4	4.7	4.2
Asthma history (%) ^a^	2.3	1.1	1.8
Allergies history (%) ^b^^,^ ^d^	6.2	3.2	4.1
Hepatitis B infection history (%)	0.2	0	0
Family history of serious infectious diseases (% yes)	4.0	3.4	5.1
Tobacco smoker (% yes)	0.2	0.9	0.6
Stressful event in last year (%) ^b^^,^ ^d^	11.6	4.2	6.7
2 or more URIs in last year (%) ^b^^,^ ^d^	20.5	11.4	12.5
Alcohol in last 30 days (% yes) ^f^	5.4	3.9	2.7
Child under 5 in home (% yes) ^c^	15.2	12.9	18.1
BCG vaccination in past (% yes) ^a^^,^ ^c^	91.6	88.1	87.8
Influenza vaccination in in year^4^ (% yes)	0.8	1.3	2.6
Covid vaccination ^e^^,^ ^f^	75.7	71.4	60.9
Dengue history ^d^	2.9	2.2	1.4
Influenza in last year (% yes)	5.1	3.6	4.0
Surgical procedure in last year ^f^ (% yes)	3.2	4.3	15.0
General anaesthetic in last year ^f^ (% yes)	0.8	1.5	6.3
Strep throat last year (%) ^e^^,^ ^f^	12.3	3.9	5.1
Physical activity <3h/week (usual level before Covid infection) ^e^^,^ ^f^	24.9	39.7	38.1

^a^ Statistically significant difference between group 1 and 2 @ p = 0.05.

^b^ Statistically significant difference between group 1 and 2 @ p = 0.01.

^c^ Statistically significant difference between group 1 and 3 @ p = 0.05.

^d^ Statistically significant difference between group 1 and 3 @ p = 0.01.

^e^ Statistically significant difference between group 1 and 2 @p = 0.001.

^f^ Statistically significant difference between group 1 and 3 @p = 0.001.

Unrecovered women are statistically different from the test-negative and reportedly SARS-CoV-2 infection history-free women for all of the characteristics listed in the preceding paragraph except asthma, and in having had more frequent dengue, alcohol consumption in the last 30 days and hypertension histories, and fewer pregnancies in the previous year, more frequent history of menstrual cycles in the preceding 3 months, and less frequent surgical and general anesthetic procedures in the last year (Table 1). Diabetes, asthma, and three BMI measures did not differ between these groups.

Covid-19 immunization was more frequent in unrecovered women than in either recovered or test negative women. Table 2 shows the detailed data about symptoms in the 630 test-positive unrecovered women. Higher frequencies of worst pain, fatigue, shortness of breath, poor sleep and difficulty remembering are seen. The low frequencies of self-reported depression, anxiety, chills, or fever, light-headedness or dizziness, cough, and changes in taste and smell are notable.

**Table 2:** Presence and intensity of 22 symptoms at a median of 6 months from diagnosis in 630 women self-assessed to be incompletely recovered from SARS-CoV-2 infection.

Symptoms	0	1	2	3	4	5
Worst pain/ache	392	46	84	63	31	14
Pain/ache locations:						
Muscles = 25						
Back/Whole body = 82						
Head = 55						
Joints = 20						
Chest = 75						
Feeling sad or depressed	594	4	11	7	10	4
Difficulty in word-finding	600	8	7	7	7	1
Light-headedness or dizziness on standing	572	9	27	13	7	2
Lack of motivation	587	6	11	18	7	1
Mental and physical fatigue/tiredness	366	25	116	79	31	13
Poor, unrefreshing sleep	564	10	27	18	7	4
Fever and/or chills	618	2	3	5	1	1
Mental confusion or disorientation	594	2	13	15	3	3
Difficulty thinking and concentrating	586	5	18	13	7	1
Shortness of breath	454	35	71	45	19	6
Reduced physical activity	576	7	25	11	6	5
Increased sensitivity to sound or light	583	7	8	15	14	3
Rapid or irregular heartbeat	581	8	14	16	7	4
Cough	593	6	17	10	3	1
Anxious or worried	571	10	16	15	12	6
Difficulty remembering	523	13	44	36	6	8
Change in sense of smell	603	6	11	6	2	2
Increased fatigue the day after more-than-usual physical or social activity	592	2	16	9	6	5
Change in sense of taste	615	3	4	6	0	2
Numbness in fingers or toes	597	9	11	9	4	0
Heat or cold intolerance	560	12	28	19	9	2

Table 3 shows the frequencies of the 6 most common symptoms in the three study groups of women. The percentages reported for each of these symptoms among the unrecovered women are significantly different from those reported by both the recovered and test-negative women at p = 0.0001. 423 (67%) of the 630 unrecovered women had at least one of the 3 most common symptoms—fatigue, pain or shortness of breath.

**Table 3:** Frequencies at a median of 6 months after SARS-CoV-2 PCR testing of the 6 most commonly reported symptoms in successfully interviewed women in 3 groups: 1) Test positive women self-assessed as unrecovered, 2) Test positive women self-assessed as recovered, and 3) Test negative women who had remained Covid-19 symptoms free.

Demographic and Medical History Characteristics	Number and % reporting symptoms		
	Group 1 Test + Unrecovered	Group 2 Test + Recovered	Group 3 Test–and Disease-Free
Total Cases	630	3102	2436
Fatigue	264 (41.9)	39 (1.3)	86 (3.5)
Pain	238 (37.8)	39 (1.3)	96 (3.9)
Shortness of breath	176 (27.9)	22 (0.7)	40 (1.6)
Difficulty remembering	107 (17.0)	25 (0.8)	37 (1.5)
Heat or cold intolerance	70 (11.1)	9 (0.3)	20 (0.8)
Poor, unrefreshing sleep	66 (10.5)	15 (0.5)	37 (1.5)

Table 4 shows the responses to questions about activities of daily living for the 3 studied groups of women. The unrecovered women report more frequent health problem- interference with their usual activities, but the percentages reporting these problems in the unrecovered group seem remarkably low compared with the percentages reporting symptoms in Table 3.

**Table 4:** Functional status for activities of daily living at a median of 6 months after Covid-PCR testing in successfully interviewed women in 3 groups: 1) Test positive women self-assessed as unrecovered, 2) Test positive women self-assessed as recovered, and 3) Test negative women who had remained Covid-19 symptoms free.

Demographic and Medical History Characteristics	Number and % reporting symptoms		
	Group 1 Test + Unrecovered	Group 2 Test + Recovered	Group 3 Test–and Disease-Free
Total Cases	630	3102	2436
Health Problems interfering with:			
Doing the usual work done before your Covid-19 testing 6 months ago ^a^^,^ ^b^	13 (2.4)	9 (0.3)	36 (1.7)
Walking upstairs ^a^^,^ ^b^	17 (2.1)	18 (0.7)	31 (1.4)
Doing household tasks involving lifting, carrying, or cleaning	75 (11.9)	128 (4.1)	143 (5.9)
Taking care of children or adults with health problems ^a^	15 (3.2)	35 (1.4)	37 (2.2)

^a^ Statistically significant difference between groups 1 and 2 @ p<0.05.

^b^ Statistically significant difference between groups 1 and 3 @ p<0.01Data about symptoms for test positive recovered and test negative women show no striking excesses of symptoms in the previously infected patients.

## Discussion

The principal findings from this study are:

- 16.9% or 1/6th of middle-aged Nepali women reported themselves as being unrecovered/unwell/unhealthy following their PCR test confirmed SARS-CoV-2 infections at a median of 6 months, and reported symptoms and functional status information consistent with these assessments. Both groups of demographically matched recovered and never-infected women, interviewed contemporaneously, reported low frequencies of the same major symptoms. The recovered and uninfected groups of women were very similar in their frequencies of health characteristics—hypertension, diabetes, asthma, allergies, tobacco abuse, strongly suggesting that the specific health characteristics that were different in the unrecovered women are genuinely associated with this condition. These data indicate that non-recovery and chronic illness after 6 months is a consequence of SARS-CoV-2 infection. The 6-month timepoint defining such illness is the metric used to diagnose chronic fatigue syndrome [14].
- The health characteristics data suggest a rich picture of immune, metabolic, and hormonal factors associated with persistence of symptoms and unrecovered status. Previously suggested increased frequencies of histories of asthma and allergies were found, but also greater immune system activation or susceptibility histories of dengue, and more frequent strep throat and URIs in the last year, and more frequent BCG vaccination were reported. While BCG vaccination has been suggested to produce protective “trained immunity” beneficial in reducing the severity of Covid-19 illness, a randomized trial of BCG vaccination in health workers to protect against Covid-19 found no evidence of benefit, with a trend suggesting increased risk of infection from this vaccination [18,19]. In the data reported here, BCG vaccination was associated with increased risk for non-recovery at 6 months. Covid vaccination history was more commonly reported in unrecovered women, but absent further details, a cause-and-effect relationship cannot be suggested.

Important and significant metabolic factor differences were reported by the unrecovered women with more frequent reported weight losses and gains of greater than 5 kg. in the period since diagnosis, and more frequent major stressful events in the previous year. Further, it is notable that in these Nepali populations, hypertension, diabetes, and particularly BMI/obesity differences between recovered and unrecovered patients were not observed.

- Some of the differences between unrecovered women and women who never developed SARS-CoV-2 infection are notable. The unrecovered women had less frequent pregnancies in the previous year, more frequent history of menstrual cycles in the preceding 3 months, and less frequent surgical and general anesthetic procedures in the last year, as well as more frequent reported weight losses and gains of greater than 5 kg. in the period since diagnosis, and more frequent major stressful events in the previous year.

These conclusions should be interpreted in the following contexts. First, the women studied were a convenience sample of individuals living in the Kathmandu valley, who self-referred themselves for SARS-CoV-2 PCR blood testing because of symptoms of infection. Thus, this study group is not randomly selected from the total Nepali population of symptom and symptomless individuals and individuals capable and incapable of seeking testing. Further, few of the studied women were likely to have been hospitalized with Covid and have incurred greater specific organ injuries of lung, heart, brain, and blood coagulation tissues and systems. Severity of illness was not otherwise assessed. During this period, the local hospitalization rates for women with Covid-19 were under 5% and limited population variant testing suggested that the prominent variants were alpha variant and delta variant [20]. The studied women were mostly urban area residents, in a country where 80% of inhabitants live in rural areas, and were likely better educated, generally younger, and healthier at the time of diagnosis than their fellow women countrywomen. These considerations signify that the studied populations are selected and are not representative of the total population of Nepal.

The details of how items are phrased in Nepali may be critical in some circumstances. Finally, we were able to contact and successfully obtain data from 59% of test positive and 42% of test negative cases or family members. While fractions of the non-recruited women were because of specific study eligibility criteria, larger proportions were non-recruited because of the specific operational conditions of the study—interviews solicited and conducted by telephone, the process of acquiring informed consent, the absence of incentives, and the time commitment. These non-interviewed women were demographically similar to those who were successfully interviewed.

The strengths of this study lie in the large numbers of women from three testing sites studied, and in the facts and circumstances that: 1. A control group of SARS-CoV-2 test negative and by history never-affected women was studied; these women reported low levels of any symptoms, with much lower frequencies than the unrecovered women, which data offer compelling evidence that the association of perceived unrecovered/unwell/unhealthy status and symptoms with SARS-CoV-2 infection is strong. 2. Assessment of recovered status was made by probing questioning about non-recovery (implying a chronic situation), presence of unwellness, and persistent perceived adverse change in health status (unhealthy) following SARS-CoV-2 infection, and symptom data were obtained directly from the patients themselves. 3. The case status was defined by a laboratory PCR test. 4. The symptoms’ descriptions were for periods of 3 days. And: 5. The symptom questionnaire had reliability and validity information suggesting reasonable credibility for the study population investigated, and the findings are internally consistent—the non-recovered patients clearly report more specific symptoms of important intensities and associated interference with activities of daily living (Tables 2 and 4) [16]. These strengths all support an argument that these observational data for the populations studied are not biased and are of high quality [21]. Finally, these data are important because they describe illness experience in women from a low-middle income country where the frequency of symptomatic and serious illness with SARS-CoV-2 has been suggested to be significantly lower than has been observed in high-income countries, and multiple confounding factors such as lower self-reported levels of depression and anxiety, less mood-altering and aspirin drug use, and lower alcohol consumption are not present [22].

Comparing the literature regarding nonrecovery and symptoms 6 months following confirmed Covid infection is problematic because the majority of reports address patients from western countries (only 2 of 9 studies in a systematic review -reference #4- had patients from non-western countries), concern previously hospitalized patients with severe acute disease, are of older patient groups, and with the exception of a recent study in 3762 volunteers, are small [1,4,23]. While the current report is not of a population-based sample, it comes closer to describing what a representative group of low- and middle-income country Covid sufferers are likely to be experiencing 6 months after their diagnoses than has been reported to date, specifically in addressing the issue of patient perceptions of their recovery status. The long term most common symptoms’ picture in the reported literature is very similar to that reported here: fatigue, CNS functional problems, and sleep disturbances [1,4,23]. Further what has been the subject of limited study to date regarding long Covid sufferers is the associated co-morbid conditions and metabolic and endocrine changes. The data reported here but begin to untangle the full spectrum of these associated factors.

### Long Covid symptoms and underlying physiologic mechanisms

Despite the richness of the reported data, the specific symptom frequencies reported by the non-recovered women do not provide themselves adequate information to propose a definition of “long-COVID” and suggest that somehow our questions missed identification of critical symptoms. Malaise, a defining symptom in chronic fatigue syndrome, was not included because of its problematic translation into Nepali [16]. Questions about energy levels, muscle weakness, appetite, and details of exercise capacities were also omitted. The assignment of non-recovery case status however, along with the specific symptoms data, validate this status assignment, and suggest metabolic and endocrine hormonal systems disruption and that future research should explore details of symptoms associated with these systems.

Importantly, these data suggest the parameters of a physiological model for the development of long- COVID similar to that proposed for chronic fatigue syndrome [14,15]. Long-COVID present at 6 months from time of infection, as seen in these Nepali women, is characterized by:

1. Immune system dysfunctional responses associated with allergies, asthma, and BCG vaccination histories; increased frequency of URIs, strep throat and dengue with neuroinflammatory symptoms of pain, difficulty remembering (suggested to be reflective of microglial or dendritic damage), and poor sleep, in the absence of fever and specific signs of active infection.
2. Metabolic and hormonal dysfunction with feelings of being unrecovered, unwell, and persistently unhealthy, which are incompletely described by usual specific symptom assessments.
3. Metabolic disturbances with specific symptoms of mental and physical fatigue, shortness of breath (without fever or cough, suggesting exercise capacity loss), pain, poor sleep, difficulty remembering, and heat and cold intolerance; and associated significant weight changes, decreased physical activity, and history of stressful events.
4. Endocrine-hormonal change hypersensitivities associated with recent pregnancy and menstrual cycling, and heat and cold intolerance.

Investigations of CFS/ME have suggested that it is a hypometabolic syndrome, and long-COVID has been hypothesized to be a one carbon stress syndrome [24,25]. Together the current and these reports suggest that long-COVID patients should be investigated for serum serine and markers of oxidative stress such as glutathione, as well as total serum B_12_, holo-transcobalamin (holoTC), the metabolic markers methylmalonic acid and homocysteine, and plasma formate. Physiological and dynamic assessment of multiple hormones are also suggested: ACTH, cortisol, TRH, TSH, thyroid, insulin, epinephrine, serotonin, melatonin, growth hormone, and aldosterone as examples [26]. A recent rigorous small study suggests that Covid infection and type 1 interferon-driven inflammation decrease serotonin levels, and that this change explains many of the major long Covid symptoms. [27] Interventions with ACTH, Vitamin B-12, folate, glutathione, and serine, directed to re-setting metabolic and hormonal systems are suggested by these interpretations and models, as have been suggested for CFS/ME [15,28].

## Conclusion

Six months after PCR test-confirmed SARS-CoV-2, 16.9% of Nepali women reported being unrecovered, and/or unwell, and/or unhealthy, with associated dominantly metabolic and hormonal systems symptoms, defining for them long-COVID. For these women immune system

over-activation factors and dysfunction were associated with this metabolic and endocrine-hormonal disruptive condition.

## Supporting information

S1 Dataset (CSV)
